# Bacterial proteins encapsulate in extracellular membrane vesicles from *Escherichia coli*-infected macrophages

**DOI:** 10.1128/mra.00020-25

**Published:** 2025-06-09

**Authors:** Risa Imamiya, Akinori Ninomiya, Hiroko Kato, Akari Shinohara, Yasuhiko Horiguchi, Mayuko Osada-Oka

**Affiliations:** 1 Food Hygiene and Environmental Health, Faculty of Life and Environmental Science, Kyoto Prefectural University 12897, Kyoto, Japan; 2 Research Institute of Microbial Diseases, Osaka University 13013, Suita, Japan; 3 Immunology Frontier Research Center, Osaka University 13013, Suita, Japan; 4 Department of Molecular Bacteriology, Research Institute of Microbial Diseases, Osaka University 13013, Suita, Japan; 5 Food Hygiene and Environmental Health, Division of Applied Life Science, Graduate School of Life and Environmental Sciences, Kyoto Prefectural University 12897, Kyoto, Japan; University of Guelph, Guelph, Ontario, Canada

**Keywords:** extracellular vesicles, exosomes, macrophage, *Escherichia coli*, outer membrane proteins

## Abstract

Extracellular membrane vesicles (EVs) are considered to be an inflammatory factor in several acute and chronic inflammatory diseases. We analyzed *Escherichia coli*-derived proteins in the EVs from macrophages that were infected with live and heat-inactivated *Escherichia coli*. Sixty-three proteins derived from live *E. coli* were detected in macrophage-derived EVs.

## ANNOUNCEMENT

We demonstrated that extracellular membrane vesicles (EVs) from macrophages infected with live *Escherichia coli* transmit proinflammatory responses to uninfected macrophages, but EVs from dead *E. coli*-infected macrophages were not inflammatory (1). Thus, proteins encapsulated in each EV were analyzed by mass spectrometry.

Mouse macrophage RAW264.7 cells (8 × 10^6^ cells) were cultured at 37°C and 5% CO_2_ for 24 h in 7 mL of Dulbecco’s modified Eagle medium (DMEM) containing 4.5 g/L glucose (Fujifilm, Osaka, Japan), 10% fetal bovine serum (FBS) (Biowest, Tokyo, Japan), 100 units/mL penicillin, and 100 mg/mL streptomycin (Gibco/Thermo, Tokyo, Japan). After changing to fresh DMEM medium without antibiotics and FBS, macrophages were incubated for 9 h at 37°C with live *E. coli* DH5α strain or *E. coli* DH5α strain inactivated by heat (75°C for 20 min). After cells were incubated with fresh DMEM medium containing antibiotics without FBS for 2 h at 37°C, the medium was again replaced with fresh DMEM medium containing antibiotics without FBS. Then, the culture medium was collected after cells were incubated for 9 h at 37°C. The culture medium was centrifuged at 2,000 × *g* at 4°C for 30 min and at 10,000 × *g* at 4°C for 30 min to remove the debris. The supernatant was filtered through a 0.2 µm filter (Millipore, Tokyo, Japan). After the filtrate was ultracentrifuged using a 50.2 Ti rotor at 100,000 × *g* at 4°C for 3 h, the pellet was ultracentrifuged at 100,000 × *g* at 4°C for 2 h with PBS. The final pellet was suspended in PBS as EVs. All the following operations were performed at 4°C. EVs were purified in at least two independent biological replicates.

The EV samples (10 µg/0.1 mL) were purified by methanol-chloroform precipitation and dissolved in 20 µL of 0.1% RapiGest (Waters, MA, USA). The protein solutions were reduced with 10 mM dithiothreitol for 70 min at 37°C and alkylated with 55 mM iodoacetamide for 70 min at room temperature under shade. The resulting protein solutions were digested with mass spectrometry-grade trypsin (Promega) overnight at 37°C. The trypsinized protein solutions were analyzed by the LC-MS/MS system consisting of HTC-PAL autosampler, the Michrom nano-Advance UHPLC (Michrom BioResources Inc., CA, USA), and LTQ Orbitrap Velos mass spectrometer (Thermo Fisher Scientific, MA, USA). MonoCapC18 High Resolution2000 (2 µm, 0.1 × 2,000 mm, GL Science, Tokyo, Japan) was employed for peptide separation. The mobile phase consisted of water containing 0.1% formic acid (solvent A) and acetonitrile (solvent B). The peptides were eluted by a linear gradient of 5%–40% B for 380 min at 500 nL/min. LTQ Orbitrap Velos was operated in the data-dependent mode to automatically switch between full scan MS and MS/MS acquisition. The mass spectrometric conditions were as follows: ion spray voltage, 1.9 kV; HCD collision energy, 35%; mass range, *m/z* 350–1,500; and dynamic exclusion, 60 s. Raw data were converted to MGF files using Proteome Discoverer 1.4 (Thermo Fisher Scientific, MA, USA) and searched against the Swiss Prot databases (*Mus musculus* and *E. coli*) by Mascot Server 2.5.1 (Matrix Science, London, UK). Precursor mass tolerance was 10 ppm, and MS/MS tolerance was 0.8 Da for Orbitrap and linear ion trap, respectively. Carbamidomethylation of cysteine, O + 18 of pyrrolysine, and iodoacetamide derivative of cysteine were specified in Mascot as fixed modifications. S-carbamoylmethylcysteine cyclization of the N-terminus, oxidation of methionine, and acetylation of the N-terminus were specified in Mascot as variable modifications. Peptide significance threshold *P* < 0.05 was used for post-search filtering. The protein threshold was set at 99.9%. The minimum number of peptides identified was set at two. Quantitative value and fold exchange were calculated by Scaffold4 (Proteome Software, USA) for MS/MS-based proteomic studies (2).

Proteomics analyses were reported in jPOST (ID: JPST003542/PXID: PXD059569). Sixty-six *E. coli*-derived proteins were detected in EVs (EC-EV) from macrophages that were infected with live *E. coli* (Table 1). In contrast, three of these proteins were exhibited in EVs (HI-EV) from macrophages that were infected with heat-inactivated *E. coli*. There were no *E. coli* proteins in EVs (n9f9-EC) from uninfected macrophages. These proteins were annotated with Gene Ontology terms, resulting in 5 plasma membrane proteins, 23 outer membrane proteins, and 22 cytoplasm proteins. At the same time, a lot of macrophage-derived proteins were detected in three kinds of EVs. These will be reported in a future issue.

**TABLE 1:** *E. coli*-derived peptide content in EVs

#	Identified proteins	Accession number	EC-EV (*n* = 2)		HI-EV (*n* = 2)		n9f9-EC (*n* = 2)	
			1	2	1	2	1	2
1	Aldehyde-alcohol dehydrogenase (adhE)	ADHE_ECO57	58	55	0	0	0	0
2	Outer membrane protein A (ompA)	OMPA_ECO57	32	45	3	2	0	0
3	60 kDa chaperonin 1 (groL1)	CH601_ECOK1	28	30	0	0	0	0
4	Periplasmic serine endoprotease DegP (degP)	DEGP_ECO57	22	14	0	0	0	0
5	Fe(3+) dicitrate transport protein FecA (fecA)	FECA_ECOLI	20	13	0	0	0	0
6	Colicin I receptor (car)	FECA_ECOLI	20	26	0	1	0	0
7	Outer membrane protein TolC (tolC)	TOLC_ECOLI	20	17	0	1	0	0
8	Uncharacterized protein YncE (yncE)	YNCE_ECOLI	19	16	0	0	0	0
9	Ferrienterobactin receptor (fepA)	FEPA_ECOLI	18	19	0	0	0	0
10	Vitamin B12 transporter BtuB (btuB)	BTUB_ECOLI	17	8	0	0	0	0
11	30S ribosomal protein S4 (rpsD)	RS4_ECO24	17	17	0	0	0	0
12	Dihydrolipoyllysine-residue acetyltransferase component of pyruvate dehydrogenase complex (aceF)	RS4_ECO24	15	11	0	0	0	0
13	30S ribosomal protein S7 (rpsG)	RS7_ECOBW	15	13	0	0	0	0
14	DNA-directed RNA polymerase subunit beta′ (rpoC)	RPOC_ECO24	14	16	1	0	0	0
15	Outer membrane protease OmpP (ompP)	OMPP_ECOLI	14	6	0	0	0	0
16	Protein TolB (tolB)	TOLB_ECO24	13	9	0	0	0	0
17	Elongation factor Tu 1 (tuf1)	EFTU1_ECO24	13	11	0	0	0	0
18	Outer membrane protein X (ompX)	OMPX_ECO57	13	9	0	1	0	0
19	Metal-binding protein ZinT (zinT)	ZINT_ECOLI	12	9	0	0	0	0
20	Major outer membrane lipoprotein (lpp)	LPP_ECO57	12	15	1	0	0	0
21	Cell division coordinator CpoB (cpoB)	CPOB_ECOLI	11	6	0	0	0	0
22	High-affinity zinc uptake system protein ZnuA (znuA)	ZNUA_ECOLI	10	10	0	0	0	0
23	Type-1 fimbrial protein, A chain (fimA)	FIMA1_ECOLI	10	12	3	4	0	0
24	Outer membrane protein assembly factor BamA (bamA)	BAMA_ECO24	8	9	0	0	0	0
25	Glyceraldehyde-3-phosphate dehydrogenase A (gapA)	G3P1_ECO57	8	4	0	0	0	0
26	30S ribosomal protein S13 (rpsM)	RS13_ECO57	8	9	0	0	0	0
27	Ribose import binding protein RbsB (rbsB)	RBSB_ECOLI	8	6	0	0	0	0
28	50S ribosomal protein L17 (rplQ)	RL17_ECO24	7	8	0	0	0	0
29	FKBP-type peptidyl-prolyl *cis-trans* isomerase FkpA (fkpA)	FKBA_ECO57	7	8	0	0	0	0
30	Putative acyl-CoA thioester hydrolase YbhC (ybhC)	YBHC_ECOLI	7	5	0	0	0	0
31	Chaperone protein Skp (skp)	SKP_ECO57	7	3	0	0	0	0
32	Outer membrane lipoprotein SlyB (slyB)	SLYB_ECO57	7	4	0	0	0	0
33	50S ribosomal protein L21 (rplU)	RL21_ECO24	7	3	0	0	0	0
34	DNA-directed RNA polymerase subunit alpha (rpoA)	RPOA_ECO24	6	11	0	1	0	0
35	Peptidoglycan-associated lipoprotein (pal)	PAL_ECO57	6	7	0	0	0	0
36	Phosphate-binding protein PstS (pstS)	PSTS_ECOLI	6	3	0	0	0	0
37	50S ribosomal protein L4 (rplD)	RL4_ECO24	6	8	0	0	0	0
38	Outer membrane protein assembly factor BamC (bamC)	BAMC_ECOLI	6	5	0	0	0	0
39	Uncharacterized protein YegR (yegR)	YEGR_ECOLI	6	2	0	0	0	0
40	Protein FimH (fimH)	FIMH_ECOLI	6	4	0	0	0	0
41	50S ribosomal protein L20 (rplT)	RL20_ECO24	5	10	0	0	0	0
42	Uncharacterized protein YqjD (yqjD)	YQJD_ECO57	5	3	0	0	0	0
43	Outer membrane protein assembly factor BamB (bamB)	BAMB_ECOLI	5	6	0	0	0	0
44	50S ribosomal protein L24 (rplX)	RL24_ECO24	5	3	0	0	0	0
45	50S ribosomal protein L2 (rplB)	RL2_ECO24	4	6	0	0	0	0
46	50S ribosomal protein L1 (rplA)	RL1_ECO27	4	7	0	0	0	0
47	Uncharacterized lipoprotein YajG (yajG)	YAJG_ECOL6	4	8	0	0	0	0
48	Penicillin-binding protein activator LpoA (lpoA)	LPOA_ECOLI	4	5	0	3	0	0
49	Rare lipoprotein A (rlpA)	RLPA_ECOLI	4	3	0	0	0	0
50	Protein YdgH (ydgH)	YDGH_ECOLI	4	2	0	0	0	0
51	50S ribosomal protein L15 (rplO)	RL15_ECO24	4	4	0	0	0	0
52	30S ribosomal protein S6 (rpsF)	RS6_ECO24	4	7	0	0	0	0
53	Outer membrane protein (ompC)	OMPC_ECOLI	3	7	0	0	0	0
54	Protein TraN GN=traN	TRAN_ECOLI	3	2	0	0	0	0
55	Chaperone protein FimC (fimC)	FIMC_ECOL6	3	2	0	0	0	0
56	30S ribosomal protein S9 (rpsI)	RS9_ECO24	3	6	0	0	0	0
57	LPS-assembly protein LptD (lptD)	LPTD_ECOK1	3	3	0	0	0	0
58	TraT complement resistance protein (traT)	TRAT1_ECOLI	3	6	0	0	0	0
59	Nucleoside-specific channel-forming protein tsx (tsx)	TSX_ECO57	3	4	0	0	0	0
60	Ferrichrome-iron receptor (fhuA)	FHUA_ECOLI	3	3	0	0	0	0
61	30S ribosomal protein S2 (rpsB)	RS2_ECO27	2	5	0	0	0	0
62	Uncharacterized protein YggE (yggE)	YGGE_ECO57	2	1	0	0	0	0
63	Inhibitor of g-type lysozyme (pliG)	PLIG_ECOLI	2	3	0	0	0	0
64	50S ribosomal protein L13 (rplM)	RL13_ECO24	2	1	0	0	0	0
65	DNA-directed RNA polymerase subunit beta (rpoB)	RPOB_ECOL5	1	4	0	0	0	0
66	50S ribosomal protein L5 (rplE)	RL5_ECO24 (+20)	1	4	0	0	0	0

## Data Availability

All data was published in jPOST under accession ID: JPST003542 (PXD059569).
